# Review of Opsoclonus-Myoclonus Ataxia Syndrome in Pediatric Patients

**DOI:** 10.3390/children11030367

**Published:** 2024-03-19

**Authors:** Mandy Hsu, Isbaah Tejani, Nidhi Shah, Rasaq Olaosebikan, Ashutosh Kumar, Sunil Naik

**Affiliations:** 1 University Park Program, Penn State College of Medicine, State College, PA 16803, USA; mhsu2@pennstatehealth.psu.edu; 2 Medical College, Aga Khan University, Karachi P.O. Box 8842, Pakistan; isbaah.tejani@scholar.aku.edu; 3 Department of Pediatric Hematology/Oncology, Penn State Health Milton S. Hershey Medical Center, Hershey, PA 17033, USA; nshah1@pennstatehealth.psu.edu (N.S.); rolaosebikan@pennstatehealth.psu.edu (R.O.); 4 Department of Pediatric Neurology, Penn State Health Milton S. Hershey Medical Center, Hershey, PA 17033, USA; akumar5@pennstatehealth.psu.edu

**Keywords:** opsoclonus-myoclonus ataxia syndrome, Kinsbourne syndrome, dancing eye dancing feet syndrome, pediatric, neuroblastoma, paraneoplastic syndrome, IVIG, rituximab

## Abstract

Opsoclonus-myoclonus ataxia syndrome (OMAS), also known as Kinsbourne syndrome, is a rare disorder that presents with myoclonus, ataxia, abnormal eye movements, irritability, and sleep disruptions, often in young children. We report a case of an infant barely 6 months old, with no significant past medical history, who presented to the emergency department with tremors, jerking motions of the head and arms, and rapid eye movements. After an extensive workup, she was found to have a neuroblastoma, which was subsequently surgically removed via thoracotomy. Despite an initial improvement in symptoms post-resection, the patient’s symptoms recurred. She was subsequently treated with dexamethasone, intravenous immunoglobulin (IVIG), and rituximab. After treatment, the patient was noted to have mild global developmental delays but was otherwise well. This case report highlights the rare occurrence of OMAS in an infant barely 6 months old at diagnosis. Using the PubMed database, a systematic review was conducted to highlight the clinical presentation, diagnosis, and management of OMAS.

## 1. Introduction

Opsoclonus-myoclonus ataxia syndrome (OMAS) is a rare central nervous system disorder with symptoms including abnormal eye movements (opsoclonus), myoclonus, and ataxia, with sleep disruptions and irritability. OMAS is also known as “dancing eye, dancing feet syndrome”, opsoclonus-myoclonus syndrome (OMS), and Kinsbourne syndrome. This syndrome occurs most commonly in young children. It is less common in children less than 6 months of age, at a prevalence of about 2% of OMAS cases [1]. The origin of OMAS is predominantly paraneoplastic and associated with neuroblastomas, or post-infectious. If a neuroblastoma is confirmed in combination with a diagnosis of OMAS, resection is often the most appropriate course of action. Immunosuppression and immunomodulation are also considered the standard of care. Immunosuppression strategies include glucocorticoids, such as intravenous (IV) methylprednisolone, or dexamethasone. Immunomodulation options include oral dexamethasone, intravenous immune globulin (IVIG), rituximab, and cyclophosphamide. Timely diagnosis is critical, along with identification of potential triggers, so that appropriate therapeutic services can be provided to support developmental milestone achievement and halt disease progression. Since children usually present with acute ataxia, differential diagnoses for OMAS may include central nervous system infection, acute cerebellar ataxia, drug intoxication, and Guillain–Barré syndrome.

## 2. Materials and Methods

In June 2023, the authors searched the scientific database PubMed using the following searches: “kinsbourne syndrome” AND “infants’, “kinsbourne syndrome” AND “pediatric”, “opsoclonus myoclonus ataxia syndrome” AND “infants”, and “opsoclonus myoclonus ataxia syndrome” AND “pediatric”. A total of 814 records were identified from these searches. The resulting study titles were exported to Excel and duplicates were removed, leaving a total of 312 unique titles.

**Figure 1:**
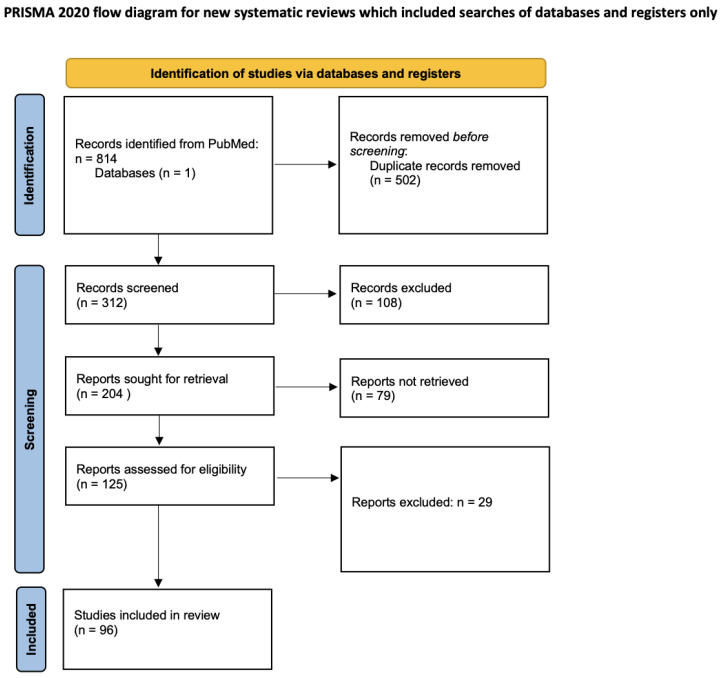
PRISMA flow chart of the screening process.

All remaining unique titles, abstracts, and full-text articles were screened by the authors for eligibility. Titles were typically excluded if they were not available in English or not relevant to OMAS and pediatric populations. Articles that were relevant to paraneoplastic syndromes and the clinical presentation, treatment, management, and developmental consequences of OMAS were included. A total of 204 articles remained following this initial screening. The authors then screened these articles by abstract. After further review, abstracts were excluded if they were not relevant to the topic, outdated, or not available in English. With these reasons for exclusion, 79 abstracts were excluded, leaving a total of 125 articles for full-text review. After screening the full-text articles for relevance, 96 articles were included for final review (see Figure 1). The information in the final articles included was then organized and presented as a review with the following categories: overview and etiologies; diagnosis, clinical presentation, and screening; treatment; and prognosis. These findings were also compared to the disease course, treatment, and management in our case report of a 6-month-old infant with OMAS.

### Case Report

We report a case of a 6-month-old infant who was seen at the Penn State Children’s Hospital, Hershey Medical Center, in December 2022.

A 6-month-old female infant with no significant past medical history presented to the emergency department for worsening tremors, jerking motions of the head and arms, and bilateral dancing-like movements in her eyes. These eye movements were reported to have started in her right eye three days prior. She was also irritable and having difficulties falling asleep. No focal seizure activity or general tonic clonic jerks in other extremities were present two days prior to the emergency room visit. Multifocal bilateral rhythmic jerking of the arms and legs began one day prior to admission. The patient never lost consciousness during any of these episodes. Her mother also noted that, about one month prior, the patient was sick with respiratory syncytial virus (RSV), which self-resolved after a one-day fever. Shortly prior to her presentation in the emergency department, the patient started losing motor milestones, such as losing the ability to roll. She was born at 37 weeks via C-section due to gestational diabetes and had one low glucose reading after birth that did not require a neonatal intensive care unit stay or intravenous fluids. Before to the onset of her symptoms, she had met all developmental milestones and was not receiving any occupational therapy, physical therapy, or speech therapy services. She had no familial history of any neurological or ocular conditions.

The initial examination in the emergency department revealed that the patient had bilateral opsoclonus, rhythmic jerking of the upper and lower extremities bilaterally, head tilting to the right, and, at times, abnormal darting tongue movements. On neurological examination, she was awake and alert, with multifocal debilitating myoclonus, bilateral opsoclonus, and bilateral ataxia. Her pupils were equal bilaterally but sluggish in reacting to light. Her vital signs were stable and she was feeding well. A respiratory virus panel, complete blood count (CBC) with differential, and complete metabolic panel (CMP) were unremarkable. Cerebrospinal fluid (CSF) analysis including an autoimmune encephalitis panel and CSF neurotransmitter metabolites was negative. The patient was then admitted to the pediatric service for further evaluation for opsoclonus myoclonus.

Upon evaluation by pediatric neurology and ophthalmology, the bilateral opsoclonus, multifocal upper and lower extremity myoclonus, and upper truncal myoclonus were confirmed. Pediatric ophthalmology found that the patient had high-amplitude movements in all directions in both eyes, but that these movements seemed to resolve while she was sleeping. On the visual acuity exam, the patient was also unable to maintain steady fixation on a stimulus due to her abnormal eye movements. Pediatric hematology/oncology was consulted for paraneoplastic concerns. A routine EEG was normal and captured opsoclonus movements that were nonepileptic. A chest CT revealed a left-sided posterior mediastinal mass less than 5 cm, supporting a diagnosis of a neuroblastoma. There was no obvious compression of any vital structures, including the heart and lungs. A follow-up MRI of the chest revealed a left paraspinal retrocrural soft tissue mass of 3.7 × 2 × 4 cm that extended behind the aorta and across the midline into the outer aspect of thoracic spine level T10, without intraspinal extension. The patient’s neck and thorax CT scans were unremarkable. The MRI of the brain, orbits, cervical spine, thoracic spine, and lumbosacral spine with and without contrast were also all normal. No metastatic lesions were found on imaging. A metaiodobenzylguanidine (MIBG) scan confirmed localized disease.

The patient was discharged and then returned to the hospital a couple of days later to undergo a posterior left thoracotomy and resection to remove the neuroblastoma. Approximately 95% tumor resection was achieved during this procedure. Biopsy samples were retrieved during surgery, which revealed small round blue cells consistent with a poorly differentiated neuroblastoma on pathology. A molecular study revealed that the MYCN gene was not amplified and that the ploidy was 1.3 with no chromosomal aberrations, placing the patient in Stage L2 of the International Neuroblastoma Risk Group (INRG) classification. Thus, at this time, chemotherapy and radiation were not recommended for our patient given her low risk status. Her opsoclonus and myoclonus significantly improved following the surgery; however, she still had some residual opsoclonus. The observation plan moving forward included urine catecholamines six weeks post-operation, outpatient follow-up with pediatric neurology and hematology/oncology, and MIBG if there was any concern of relapse or recurrence. Furthermore, she would require regular imaging, laboratory assessments, and physical exams to monitor her condition at periodic intervals. 

At one-week post-resection, she was able to maintain fixation on an object without having bouncing eyes. However, about two weeks after the surgical resection, the patient was noted to have worsening myoclonus, sleep difficulties, and irritability. Therefore, she was admitted to the hospital for IVIG 1 g/kg/day for two days and IV methylprednisolone 30 mg/kg/day for five days, to continue treating her opsoclonus myoclonus. Clonazepam 0.25 mg was given for symptomatic relief from the myoclonus. She was discharged with an outpatient therapy regimen of dexamethasone for three days and IVIG 1 g/kg on day three, with ongoing treatment every month for a year. She received four rituximab infusions of 375 mg/m^2^/dose, which she tolerated without difficulty or signs of reaction. The eight-week postoperative MRI showed no evidence of residual disease, recurrence, or metastasis, with an improvement in the opsoclonus-myoclonus symptoms. 

At 6 months post-surgical resection, no nystagmus or nystagmoid eye movements were seen by ophthalmology. There was no concern for recurrent opsoclonus on exam. The patient’s family was also counseled about monitoring for increased intraocular pressure secondary to receiving dexamethasone treatment. At 9 months post-surgical resection, the patient was recovering well and no longer experienced any irritability, sleep concerns, or myoclonus. Her follow-up MRIs of the abdomen continued to show no evidence of recurrence of the resected neuroblastoma or metastasis. The follow-up brain MRI at one year post-resection showed a new nonspecific, non-enhancing 7 × 6 mm focus of abnormal signal intensity in the posterior limb of the left internal capsule. This finding did not appear to be a metastatic tumor and may have been the result of a prior infection, inflammation, or demyelination that may or may not have been related to her prior OMAS. A follow-up of the brain MRI was scheduled for the upcoming 3–6 months to continue monitoring the stability of this new development. 

This patient still currently has a few gross and fine motor delays. She continues to receive regular physical therapy and occupational therapy to manage these delays. Following a year of treatment, she will receive maintenance doses of IVIG once every two months two times, and then every three months two times for a total duration of 10 months. Given that there is no consensus standard of care for OMAS treatment at present, this treatment plan was agreed upon by a multidisciplinary team. This team consists of pediatric ophthalmology, hematology/oncology, neurology at multiple institutions, and the patient’s family. A few considerations when making this treatment plan included the patient’s previous favorable response to dexamethasone and IVIG, as well as her insurance coverage.

## 3. Review

### 3.1. Overview and Etiologies

OMAS is a rare syndrome that presents most commonly in children. Pang et al. conducted a prospective study that reported the incidence of OMAS as 0.18 cases/million [2], while Hasegawa et al. reported an estimated incidence of 0.27–0.40 cases/million [3]. The median age of disease onset is 18 months with a range of 3 months to 8.9 years [4].

While OMAS occurs in only 2–3% of those with neuroblastoma, around 50% of children who present with OMAS are eventually diagnosed with neuroblastoma [5]. Neuroblastomas that are associated with OMAS are usually small, in the early INRG stages (Stages I–II), and with no MYCN gene amplification or metastases [5,6]. Typically, neuroblastomas can be found in the adrenal glands, but, since these tumors may arise from any neural crest cells in the body, some literature has reported OMAS-related neuroblastomas in rarer locations, such as the pancreas [7]. While neuroblastoma is the most common tumor type (73%), other tumors associated with OMAS include ganglioneuroblastoma (22%) and ganglioneuroma (4%) [8]. Due to its association with neuroblastomas and related tumors, OMAS is considered a paraneoplastic phenomenon. Its pathophysiology has been suggested to involve autoreactive T-cell activation, B-cell activation, and the presence of a variety of autoantibodies, such as anti-Hu antibodies, anti α-enolase antibodies, and autoantibodies against cerebellar granular and Purkinje cells [9,10,11]; however, the pathogenesis of OMAS is not yet completely understood.

OMAS also has a para-infectious etiology; studies have reported its association with Epstein–Barr virus [12], hepatitis C virus [13], rotavirus [14], adenovirus [15], enterovirus [16], rhinovirus, RSV [17], human herpesvirus 6 [18], malaria [19], and COVID-19 [20]. Some studies have shown the development of OMAS to be secondary to immune reconstitution after highly active antiretroviral therapy (HAART) for HIV [21] and severe acute malnutrition [22], while its association with type 1 diabetes mellitus [23,24] and Aicardi–Goutières syndrome [25] has also been reported. 

Neurophysiological tools such as electroencephalogram (EEG), visual evoked potential (VEP), and somatosensory evoked potential (SEP) have demonstrated cerebral dysfunction in children with OMAS, suggesting cerebral cortex involvement in the pathogenesis of OMAS [26]. fMRI studies have shown greater activity between the cerebellum and visual–parietal cortices and decreased activity between the cerebellum and primary motor cortex in patients with OMAS [27]. The genomic profiling of neuroblastomas associated with OMAS showed both segmental and numerical chromosome alterations, although these profiles were not related to the patient’s prognosis [28].

### 3.2. Diagnosis, Clinical Presentation, and Screening

A diagnosis of OMAS can be made if three of the following four features are present in a patient: (1) opsoclonus, (2) ataxia or myoclonus, (3) behavior changes or sleep disturbances, and (4) neuroblastoma [8]. OMAS classically presents as opsoclonus, myoclonus, and ataxia. Children can also present with significant mood and behavioral problems, such as irritability, inconsolability, agitation, and changes in personality, along with speech and sleep disturbances, tremors, and dysmetria [3,29,30,31,32,33]. However, OMAS may present with a variety of other symptoms. For instance, less commonly reported symptoms include an augmented startle response and blink reflex [34]. Other, less common findings may include severe cerebellar atrophy and hypoperfusion [35]. Our patient presented with some of the classic signs of OMAS, which notably included severe irritability and multifocal myoclonus. However, her symptoms had an abrupt and distressing onset, to the degree that she required symptomatic treatment. Similarly to our patient, many children also experience the regression of developmental milestones and may lose acquired motor and language skills [8,36].

An atypical presentation of OMAS can lead to a misdiagnosis of cerebellar ataxia or acute cerebellitis [37,38,39], leading to a delay in diagnosis. This delay averages about 8.4 months after the initial presentation [40]. Patients may also present with florid jerking of the limbs that can resemble a tonic clonic seizure, which is sometimes misdiagnosed as epilepsy or status epilepticus. Since ataxia may precede other symptoms, acute post-infectious cerebellar ataxia is another potential differential diagnosis [41]. At times, some symptoms may even be transient and self-resolve gradually [42]. Cases have been reported wherein such patients were started on anti-epileptic medications, without any improvement in their symptoms, and were eventually diagnosed with OMAS [43,44]. Thus, it is important to distinguish OMAS from other conditions that might present with similar symptoms, so that appropriate treatment may be started in a timely manner. 

Although the diagnosis of OMAS is clinical, certain laboratory tests are usually performed to rule out other neurological and inflammatory conditions. These tests also establish the etiology, which is particularly important because of the association between OMAS and neuroblastoma [8]. Routine tests such as a CBC, renal and liver function tests, clotting studies, and inflammatory markers (erythrocyte sedimentation rate and C reactive protein) are typically obtained [8]. A lumbar puncture followed by the analysis of CSF is also usually performed, which can show elevated white blood cell counts and the presence of oligoclonal bands [1,37,45]. Recent studies have also shown B-cell expansion in the CSF of OMAS patients [46,47]. Pranzatelli et al. reported that B-cells increased as the disease severity (marked by motor impairment) increased and decreased with the disease duration, which makes it an effective biomarker of active OMAS [48]. If an infectious etiology such as mycoplasma is suspected, PCR and/or serology of blood, CSF, or nasopharyngeal aspirates can be performed [8]. 

To screen OMAS patients for neuroblastoma, imaging modalities such as whole-body CT, MRI, or MIBG scintigraphy should be applied [37,49]. Urine catecholamine metabolites and specific autoantibodies associated with OMAS and neuroblastoma can also be measured [8,37]. Brunklaus et al. reported that CT/MRI imaging of the chest and abdomen was the most accurate tool to detect occult neuroblastoma, while MIBG and urine catecholamines were less sensitive, especially in tumors with low metabolic activity [50]. In contrast, Swart et al. reported a case study of a 13-month-old girl for whom MIBG scintigraphy was successful in detecting a neuroblastoma, while all other imaging tests, including chest X-ray and abdomen ultrasound, were normal [51]. Our patient received the majority of these screening modalities, including CT and MRI of multiple areas, as well as urine catecholamine metabolite measurements and MIBG scintigraphy. All of these evaluations added to our clinical picture, the eventual diagnosis of OMAS, and the treatment considerations. ^68^Ga-labeled DOTANOC PET/CT, a newer imaging technique, has also shown high diagnostic accuracy for neuroblastic tumors within a small sample of OMAS patients [52]. 

### 3.3. Treatment

Multiple treatment modalities are used for OMAS, with no specific treatment having shown reliably consistent resolution of all symptoms [37]. However, early treatment is indicated and has been shown to be associated with better long-term outcomes [53]. Mizia-Malarz et al. found that long-term sequelae occurred in all patients that were treated late, but only in 42% of patients that were treated early [54]. In some cases whereby OMAS is associated with neuroblastoma, tumor resection has led to the alleviation of symptoms, but, in others, the symptoms persisted [30,37,55]. Adrenocorticotropic hormone (ACTH) [54], steroid therapies [56,57], and IVIG are commonly used in the treatment of OMAS. A randomized controlled trial of 53 participants demonstrated a significantly superior response in patients who were given a combination of IVIG and prednisolone as compared to prednisolone alone [58]. Newer drugs, such as rituximab, are also now being used in the treatment of OMAS, with positive results [59,60,61,62,63]. Rituximab has been used as an adjunct therapy to steroids or ACTH, causing B-cell depletion in the CSF [64,65,66]. It is also used to treat relapse [67]. Immunomodulatory agents such as cyclophosphamide [68,69], 6-mercaptopurine [70], and azathioprine [37] are also being used. Plasmapheresis has been used for the treatment of OMAS in cases where the more conventional therapies have failed, and it has proven to be successful, as it removes autoantibodies from circulation [71,72,73]. Autologous cell transplantation therapy has been reported in two patients, in which one had complete resolution while the other had minimal changes in symptoms. The rationale is that although the etiology of OMAS is not completely understood, it may be an autoimmune paraneoplastic disorder with neuroinflammation. Therefore, removing pathogenic autoreactive cells and replacing them with normal immune cells would help to improve the symptoms [74]. Certain drugs, such as clonazepam [75], have also been used to successfully improve the symptoms of OMAS in some patients. Ofatumumab [76,77] has also been used to treat OMAS, and there are some rare reports of patients with the spontaneous resolution of OMAS symptoms without any immunotherapies, followed by normal growth and development [78]. Overall, multimodal or combination therapies are typically used in the treatment of OMAS [79]. Studies conducted to compare the safety and efficacy of combination therapies have shown that corticotropin- [80] or dexamethasone-based combination immunotherapies have greater efficacy as compared to the conventional monotherapy of using corticotropin or dexamethasone alone [81]. Of note, although anticholinergic drugs suppress acetylcholine release and may be used to treat myoclonus symptoms, two pediatric case studies of OMAS patients demonstrated the exacerbation of the involuntary OMAS symptoms when atropine was used with other sedatives [82]. Our case report patient had modest to moderate improvement after IVIG until rituximab was given, which led to a significant improvement. Additionally, for our patient, high-dose dexamethasone and IVIG were critical in helping to keep her in remission, highlighting the importance of these post-surgical outpatient follow-up treatments. Due to the lack of consensus regarding how to treat OMAS, a variety of treatments may need to be trialed to effectively address the symptoms. From our case report, we have also learned that other institutional considerations, such as the availability of medications, insurance coverage, and scheduling concerns, may also play a role in devising an appropriate treatment plan. 

### 3.4. Prognosis

Neuroblastomas associated with OMAS have a favorable prognosis as these tumors are usually small and well differentiated, leading to a significantly higher rate of survival [37,83,84]. Additionally, OMAS patients with mediastinal neuroblastomas are associated with better clinical presentations and outcomes than OMAS patients with non-mediastinal neuroblastomas [85]. Although our patient had a mediastinal neuroblastoma, it was 4 cm in size and located extremely close to the spine. Furthermore, it was a probable congenital tumor with aggressive growth to 4 cm over 6 months, as there was no other evidence of or inciting factors for tumor formation post-birth. Therefore, timely workup and treatment were especially important for her prognosis. While the symptoms of OMAS may resolve with time and appropriate treatment, neurological, behavioral, and cognitive deficits are common in patients [86,87,88,89]. Sun et al., in a retrospective review of 14 children with OMAS and neuroblastoma, reported that approximately 93% children had some neurological sequelae, with behavioral changes being the most common, followed by language impairments, motor retardation, and cognitive disorders. They also reported that the risk factors for neurological sequelae were the female sex, a residual tumor, and a non-adrenal-gland-located tumor [90]. Goh et al. followed three children with OMAS from 4 to 10 years of age and reported that significant deficits in attention, processing speed, visuospatial skills, and language were observed in the long term [91]. In 2011, Brunklaus et al. concluded, from a retrospective review of 101 patients with OMAS over a 53-year period, that 60% of the patients had residual motor problems, while 66% had speech abnormalities, 51% had learning disabilities, and 46% had behavioral problems. One third of these patients had normal intellectual outcomes and cessation of symptoms. Those with cognitive impairments had a severe initial presentation, were younger at disease onset, and had a chronic-relapsing disease course [4]. Our patient did not experience any delays until the onset of her OMAS symptoms, and these delays were limited to her motor development. Following her resection and treatment, she regained the motor milestones that she had lost but still had motor development delays, which require ongoing physical and occupational therapies. Her speech and social milestones remained unaffected throughout this course. Other presentations and outcomes were reported by Green et al., who found auditory hypersensitivity and other atypical sensory symptoms as long-term consequences of OMAS [92]. No significant differences in neurological outcomes were reported in Krug et al.’s study comparing the long-term sequelae between OMAS patients who did and did not have a neuroblastoma [93]. Apart from neurological sequelae, these patients may also be at risk of developing new paraneoplastic symptoms in the long term, as described in Morales La Madrid et al.’s case report of a child who developed limbic encephalitis associated with anti-Hu antibodies 6 years after the diagnosis of neuroblastoma and OMAS [94].

Recognizing and treating relapse is especially important for OMAS, as it has implications for patients’ quality of life and complications. Relapse can appear as the abrupt reappearance of OMAS symptoms in children who have previously demonstrated the remission of symptoms, although these symptoms may vary [95]. Because of this, it is especially important to implement regularly scheduled follow-ups and monitoring with a multidisciplinary care team, which may include pediatric neurology, surgery, and ophthalmology, as well as infectious disease or hematology/oncology when appropriate. Considering our patient, follow-up imaging has been of value in detecting findings early, such as the new, small, abnormal signal intensity on the brain MRI. Detecting these changes early is necessary to establish a baseline and to monitor for adverse developments. Our patient also continues to receive regular MRIs of the abdomen and chest, as well as lab screening to monitor for neuroblastoma recurrence or evidence of metastasis. Given that the pathogenesis and treatment of OMAS are not yet completely understood, the main course of treatment for relapse is through prevention approaches such as these. Corticosteroids and IVIG as treatments cannot completely prevent relapses from occurring. In these situations, it is suggested to consider using a multimodal combination immunotherapy, including disease-modifying agents, early on to improve the long-term outcomes, when the benefits may outweigh the risks of immunotherapy [94].

## 4. Conclusions

OMAS, also known as OMS and Kinsbourne syndrome, is a rare neurological disorder characterized by abnormal eye movements, myoclonus, and ataxia, along with behavioral abnormalities and sleep disturbances. It most commonly occurs in children, but it is very rare in infants less than 6 months of age. Our case report describes one of these rare instances of OMAS in a young patient. Her symptom onset, presentation, diagnosis, and treatment course provide an example of how OMAS associated with a paraneoplastic neuroblastoma in young infants may be managed. Half of the cases that present in children are associated with neuroblastomas, but other idiopathic causes of OMAS may be of viral origin. Although OMAS is mostly a clinical diagnosis, tests and imaging such as lumbar puncture, CT, MRI, and urine tests are helpful, especially when monitoring for post-surgical recurrence when a paraneoplastic neuroblastoma is involved. This is especially evident in the case of our patient. There is not yet one specific treatment that reliably demonstrates improvements in symptoms; however, surgical resection and immunosuppressive therapy are most often used and have demonstrated success in the existing literature on OMAS. For our patient, a combination of surgical resection, immunosuppressive therapy, IVIG, and glucocorticoids was the most successful.

Authors’ Note: Families can search for further information about OMAS through the OMS Life Foundation and Dancing Eye Syndrome Support Trust.
